# Evolution of seroprevalence to SARS‐CoV‐2 in blood donors in Sarajevo Canton, Federation of Bosnia and Herzegovina: Cross‐sectional and longitudinal studies

**DOI:** 10.1111/irv.13182

**Published:** 2023-08-22

**Authors:** Sanjin Musa, Elma Catovic Baralija, Veronica Ivey Sawin, Anthony Nardone, Mirza Palo, Sinisa Skocibusic, Mia Blazevic, Seila Cilovic Lagarija, Gorana Ahmetovic‐Karic, Alma Ljuca, Sanela Dostovic‐Halilovic, Rozalija Nedic, Lorenzo Subissi, Rawi Ibrahim, Golubinka Boshevska, Isabel Bergeri, Richard Pebody, Aisling Vaughan

**Affiliations:** ^1^ Institute for Public Health of the Federation of Bosnia and Herzegovina Sarajevo Bosnia and Herzegovina; ^2^ Sarajevo School of Science and Technology Sarajevo Medical School Sarajevo Bosnia and Herzegovina; ^3^ Institute for Transfusion Medicine of the Federation of Bosnia and Herzegovina Sarajevo Bosnia and Herzegovina; ^4^ Epiconcept Paris France; ^5^ World Health Organization Office in Bosnia and Herzegovina Sarajevo Bosnia and Herzegovina; ^6^ World Health Organization Geneva Switzerland; ^7^ World Health Organization Regional Office for Europe Copenhagen Denmark

**Keywords:** Bosnia and Herzegovina, cross‐sectional study, longitudinal study, SARS‐CoV‐2, seroprevalence

## Abstract

**Background:**

Sarajevo Canton in the Federation of Bosnia and Herzegovina has recorded several waves of high SARS‐CoV‐2 transmission and has struggled to reach adequate vaccination coverage. We describe the evolution of infection‐ and vaccine‐induced SARS‐CoV‐2 antibody response and persistence.

**Methods:**

We conducted repeated cross‐sectional analyses of blood donors aged 18–65 years in Sarajevo Canton in November–December 2020 and 2021. We analyzed serum samples for anti‐nucleocapsid (anti‐N) and anti‐spike (anti‐S) antibodies. To assess immune durability, we conducted longitudinal analyses of seropositive participants at 6 and 12 months.

**Results:**

One thousand fifteen participants were included in Phase 1 (November–December 2020) and 1152 in Phase 2 (November–December 2021). Seroprevalence increased significantly from 19.2% (95% CI: 17.2%–21.4%) in Phase 1 to 91.6% (95% CI: 89.8%–93.1%) in Phase 2. Anti‐S IgG titers were significantly higher among vaccinated (58.5%) than unvaccinated infected participants across vaccine products (*p* < 0.001), though highest among those who received an mRNA vaccine. At 6 months, 78/82 (95.1%) participants maintained anti‐spike seropositivity; at 12 months, 58/58 (100.0%) participants were seropositive, and 33 (56.9%) had completed the primary vaccine series within 6 months. Among 11 unvaccinated participants who were not re‐infected at 12 months, anti‐S IgG declined from median 770.1 (IQR 615.0–1321.7) to 290.8 (IQR 175.7–400.3). Anti‐N IgG antibodies waned earlier, from 35.4% seropositive at 6 months to 24.1% at 12 months.

**Conclusions:**

SARS‐CoV‐2 seroprevalence increased significantly over 12 months from end of 2020 to end of 2021. Although individuals with previous infection may have residual protection, COVID‐19 vaccination is vital to strengthening population immunity.

## 1 INTRODUCTION

Within 2 years following the World Health Organization's (WHO) declaration of the novel coronavirus disease (COVID‐19) as a public health emergency of international concern,^1^ a significant proportion of the world's population developed an immune response to severe acute respiratory syndrome coronavirus 2 (SARS‐CoV‐2), via infection and or vaccination.^2^

Bosnia and Herzegovina, a country in the Western Balkan region with 3.3 million residents, is composed of two entities: the Federation of Bosnia and Herzegovina (FBiH) and the Republika Srpska (RS). Sarajevo Canton (population 419,918) is one of 10 cantons in the FBiH, which includes City of Sarajevo, the capital and largest city in Bosnia and Herzegovina.^3^ Sarajevo Canton has recorded several waves of high SARS‐CoV‐2 transmission and has struggled to attain adequate vaccination coverage. As of 31 January, 2022, BiH had recorded 368,564 cases and 14,481 deaths associated with COVID‐19, with 92,344 cases and 1745 deaths in Sarajevo Canton.^4^

COVID‐19 vaccines first arrived in Sarajevo Canton in March 2021, though uptake was stymied by limited supplies and vaccine hesitancy. By the end of 2021, only 27.8% of residents aged 18 years or older in the FBiH and 41.3% in Sarajevo Canton had received at least one dose.^4^ However, official data likely underestimate vaccination coverage due to vaccination of BiH residents in neighboring countries. The Institute for Public Health (IPH) of Sarajevo Canton estimates 54.3% of residents are fully vaccinated, including those who voluntarily registered their vaccination in neighboring countries.^5^ Vaccines administered in the FBiH include Comirnaty (Pfizer‐BioNTech), AstraZeneca, Sinopharm, CoronaVac (Sinovac), and SpikeVax (Moderna).^6^

Serosurveys provide critical estimates of population immunity, particularly in areas with suboptimal disease surveillance and vaccine coverage.^7^ Asymptomatic or mild disease is common, which leads to a high prevalence of under‐detection.^8^, ^9^ Although knowledge of the virological, epidemiological, and clinical characteristics of SARS‐CoV‐2 has progressed considerably since the beginning of the pandemic, few studies have been conducted in the Western Balkan region.

Using an adapted WHO UNITY study seroepidemiological investigation protocol,^10^ we aimed to estimate the seroprevalence of anti‐SARS‐CoV‐2 antibodies in Sarajevo Canton by conducting repeated cross‐sectional analyses among blood donors (aged 18–65 years) at 12‐month intervals from end of 2020 to end of 2021; to assess immune durability at 6‐ and 12‐month intervals among those who were seropositive at end of 2020; and to describe the antibody response among blood donors with infection‐ and/or vaccine‐induced immunity, including across vaccine products. This will support improved understanding of population immunity and provide evidence to support informed public health decision making.

## 2 METHODS

### 2.1 Study design and participants

**FIGURE 1:**
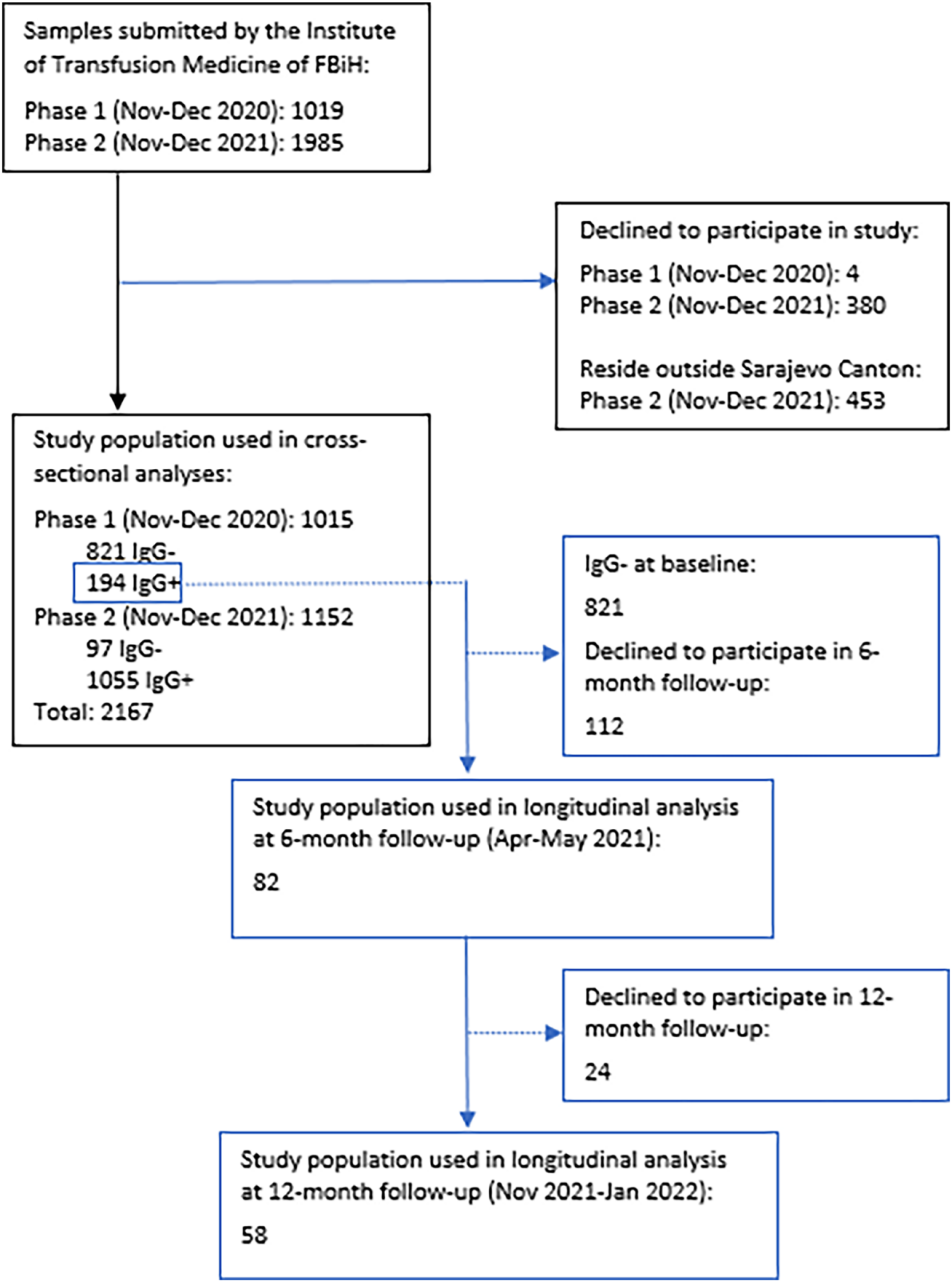
Participant flow diagram. Participants were excluded due to refusal to participate or residence outside Sarajevo Canton.

We conducted repeated cross‐sectional analyses among blood donors aged 18 to 65 years who attended the Institute of Transfusion Medicine of the FBiH at end of 2020 (Phase 1) and end of 2021 (Phase 2) to estimate the seroprevalence of anti‐SARS‐CoV‐2 antibodies in Sarajevo Canton. We aligned methods with WHO standardized protocol under the Unity Studies initiative.^10^ In the cross‐sectional analyses, blood donors were invited to participate in the study, irrespective of prior SARS‐CoV‐2 infection (Figure 1). Non‐residents of Sarajevo Canton and those experiencing COVID‐19 symptoms, or who reported contact with a confirmed COVID‐19 case within 14 days prior, were excluded.

In the longitudinal analysis, seropositive participants in Phase 1 were contacted via telephone and invited to participate in follow‐up assessments at approximately 6‐ and 12 months after baseline to assess immune durability. Participants were excluded if they refused to participate or were no longer residents of Sarajevo Canton.

Partially vaccinated participants, and those who had completed the primary series more than 6 months prior or had received a third booster dose were included in overall seroprevalence estimates but excluded from other analyses that aimed to estimate the effect of vaccination on population immunity.

### 2.2 History of COVID‐19 and vaccination

After providing written consent, all eligible participants completed a questionnaire regarding their history of suspected or confirmed COVID‐19 infection within the last 9 months (Phase 1) or 3 months (Phase 2), including whether they had experienced COVID‐19 symptoms, had received a diagnosis of COVID‐19, or had been hospitalized due to COVID‐19. Participants also provided their vaccination status (Phase 2) via verbal report or vaccination card. We determined individuals to be fully vaccinated against COVID‐19 if they had completed the primary vaccine series at least 14 days but no more than 180 days prior to serological testing. Vaccines were stratified by platform: mRNA vaccines (Comirnaty and SpikeVax), inactivated vaccines (Sinopharm and CoronaVac), and viral vector vaccines (AstraZeneca and Johnson & Johnson). Participants who were seropositive but reported no history of symptomatic infection, diagnosis, or vaccination were considered to have had asymptomatic infection.

Participants in the longitudinal analysis were stratified according to their exposure status over the study period, including those who reported no vaccination and their antibody titers did not increase from baseline at follow‐up visits (primarily infected), those who reported no vaccination and their antibody titers increased over follow‐up (re‐infected), and those who were fully vaccinated against COVID‐19 (vaccinated).

### 2.3 Serological testing

Trained laboratory staff at the Institute for Transfusion Medicine of the FBiH collected venous blood samples from participants and conducted laboratory testing. Plasma samples (500 μL aliquot) of seropositive participants were stored at −30°C and archived for possible additional testing for up to 2 years, as described on the informed consent forms signed by participants. Anti‐SARS‐CoV‐2 antibodies were detected by chemiluminescent microparticle immunoassays (CMIA) using the Abbot Laboratories SARS‐CoV‐2 IgG I and II kits and ARCHITECT i2000sr system.

In Phase 1, serum samples were assessed for anti‐nucleocapsid (anti‐N); 1.4 AU/mL or greater was considered seropositive for anti‐N. In Phase 2, serum samples were assessed for the receptor binding domain (RBD) of the S1 subunit of the spike protein of SARS‐CoV‐2 in serum and plasma (anti‐S); 50.0 AU/mL or greater was considered seropositive for anti‐S.

In the longitudinal analysis, anti‐N seropositive participants at baseline were contacted via telephone and invited to participate in follow‐up assessment at 6‐ and 12 months for anti‐N and anti‐S seropositivity. Using stored aliquots, we retroactively assessed anti‐S antibodies among participants who were seropositive for anti‐N at baseline and included in the 6‐month follow‐up.

### 2.4 Statistical analysis

Participants were stratified by age (18–29, 30–39, and 40–65 years) and sex. The crude seroprevalence estimates and 95% confidence intervals were adjusted using direct standardization for the age and sex distribution of Sarajevo Canton based on April 2021 data from the Office of Statistics for the FBiH.^3^ Crude and adjusted seroprevalence estimates are presented. Mean and standard deviations were computed for continuous variables, and frequency distributions were calculated for categorical variables. Chi‐squared tests were conducted on categorical variables. IgG titers were presented using median and interquartile range (IQR). Logarithmic transformation of the IgG values was used to present the distribution. Differences between groups were examined using the Wilcoxon rank‐sum test (cross‐sectional) and the Student's paired *t* test (longitudinal). The Institute of Transfusion Medicine of the FBiH typically receives 1000 to 1500 blood donations per month. Assuming a seroprevalence of 5%–50% during the study period and a 95% confidence interval, inclusion of 300–500 blood donors per age stratum would allow a 2%–6% margin of error for seroprevalence estimates. All statistical analyses were conducted in R 4.2.0.

## 3 RESULTS

### 3.1 Cross‐sectional analyses

A total of 1015 blood donors were recruited in Phase 1 (November 2–December 11, 2020) and 1152 in Phase 2 (November 7–December 31, 2021) (Figures 1 and S1). In comparison to Phase 1 (*n* = 4), there were significantly more participants who refused to participate in Phase 2 (*n* = 380). Reasons for refusal include prior knowledge of antibody status and younger donors' lack of interest in knowing their antibody status (85 high school students).

In both cross sections, the median age was 35 years (range 18–65 years), and the majority of participants were male (73.9% in Phase 1 and 66.6% in Phase 2) (Table 1). More participants reported COVID‐19 symptoms in Phase 1 (21.3%) than in Phase 2 (14.1%). History of hospitalization due to COVID‐19 was rare in both study populations (0.3% in Phase 1 and 0.1% in Phase 2).

**TABLE 1:** Demographic characteristics in study population, Sarajevo Canton, BiH, Nov/Dec 2020–Nov/Dec 2021.

	Phase 1 (Nov/Dec 2020)		Phase 2 (Nov/Dec 2021)	
	*N*	%	*N*	%
Overall	1015	100.0	1152	100.0
Age (years)	Median (range)	35 (18–65)	Median (range)	35 (18–65)
18–29	341	33.6	419	36.4
30–39	320	31.5	323	28.0
40–65	354	34.9	410	35.6
Sex				
Male	750*	73.9	767*	66.6
Female	265*	26.1	385*	33.4
History of COVID‐19 infection^a^				
Diagnosed	54	5.3	47	4.1
Symptomatic	216	21.3	163	14.1
Hospitalized	3	0.3	1	0.1
Received COVID‐19 vaccine	–	–	788	68.4
Fully vaccinated ≤6 months	–	–	675	58.6
Partially vaccinated	–	–	46	4.0
Last dose >6 months prior	–	–	64	5.6
Received booster dose	–	–	3	0.3

* Statistically significant (*p* < 0.05).

^a^ Survey included self‐reported history of COVID‐19 symptoms, diagnosis, and hospitalization over previous 9 months for Phase 1 and previous 3 months for Phase 2.

In Phase 1 of our cross‐sectional analysis, 49% of seropositive participants reported no history of symptoms associated with COVID‐19 within the previous 9 months. Of those who were asymptomatic, 62.1% were aged younger than 40 years. Among seropositive and symptomatic participants, few sought medical attention (7.1% aged 18–29 years and 8.1% aged 30–39 years).

No participants were vaccinated against COVID‐19 in Phase 1. In Phase 2, 675 participants (58.6%) were fully vaccinated, including 290 (43.0%) who had received Comirnaty, 221 AstraZeneca (32.7%), 125 Sinopharm (18.5%), 19 CoronaVac (2.8%), 15 Janssen (Johnson & Johnson) (2.2%), and 5 SpikeVax (0.7%). Vaccination status was reported verbally by 307 (45.5%) participants and with written documentation by 342 (50.7%) participants. An additional 46 participants (4.0%) were partially vaccinated, 64 (5.6%) had completed the primary series more than 6 months prior, and 3 (0.3%) had received a third dose. The elapsed time between completion of the primary COVID‐19 vaccine series and serological testing ranged from 14 to 180 days (median 106 days, IQR 65–148 days).

Population‐adjusted seroprevalence in Sarajevo Canton increased significantly from the end of 2020 to the end of 2021 (*p* < 0.001) (Table 2). In Phase 1, 194 (19.2%) of 1015 participants were positive for anti‐N IgG antibodies against SARS‐CoV‐2, with an adjusted seroprevalence of 17.7% (95% CI 15.4%–20.2%). Among seropositive participants, 99 (51.0%) reported COVID‐19 symptoms. In Phase 2, 1053 (91.6%) of 1152 participants were positive for anti‐S IgG antibodies, with an adjusted seroprevalence of 91.6% (95% CI 87.0%–96.5%).

**TABLE 2:** Crude and adjusted SARS‐CoV‐2 IgG antibody seroprevalence estimates by demographic characteristics, Sarajevo Canton, BiH, Nov/Dec 2020–Nov/Dec 2021.

	All samples	IgG+ samples	Crude seroprevalence		Sarajevo Canton population (Apr 2021)	Directly standardized seroprevalence	
	*N*	*n*	%	(95% CI)	*N*	%	(95% CI)
Phase 1 (Nov–Dec 2020)							
Overall	1015	194	19.2	17.2–21.4	273,891^a^	17.7	15.4–20.2
Age (years)							
18–29	341	70	20.5	16.4–25.2	59,869	18.3	14.0–23.5
30–39	320	58	18.1	14.1–22.8	64,559	17.3	12.4–23.8
40–65	354	66	18.6	14.7–23.1	149,463	17.6	12.6–24.3
Sex							
Male	750	157	20.9	18.1–24.0	131,032^a^	20.5	17.3–24.3
Female	265	37	14.0	10.0–18.7	142,859^a^	15.0	9.9–22.0
Phase 2 (Nov–Dec 2021)							
Overall	1152	1053	91.6	89.8–93.1	273,891^a^	91.6	87.0‐96.5
Age (years)							
18–29	419	383	91.4	88.3–93.9	59,869	90.9	81.9–100.1
30–39	323	299	92.6	89.1–95.2	64,559	93.9	82.0–107.0
40–65	410	373	91.0	87.8–93.6	149,463	90.9	80.8–102.0
Sex							
Male	767	700	91.3	88.9–93.2	131,032^a^	91.1	84.2–98.5
Female	385	355	92.2	89.1–94.7	142,859^a^	92.1	81.3–104.0

^a^ Population aged 18–65 years in Sarajevo Canton.

**FIGURE 2:**
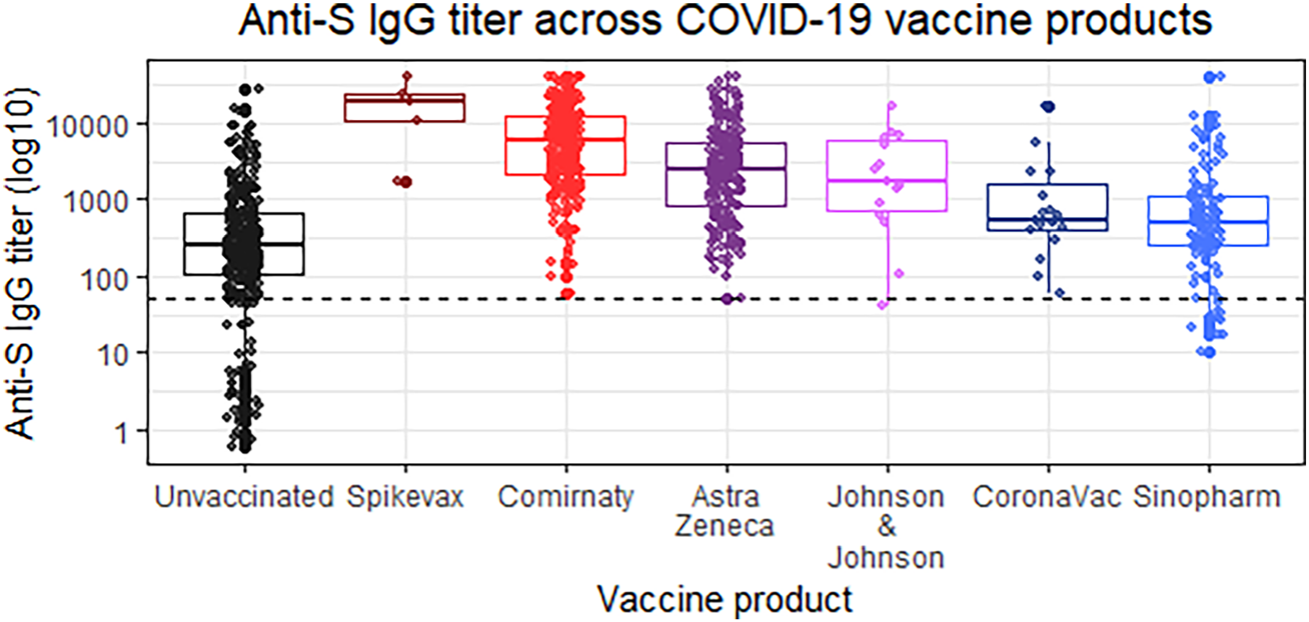
Anti‐S IgG antibody response across types of vaccine products and history of SARS‐CoV‐2 infection, Sarajevo Canton, BiH, Nov/Dec 2021 (within 180 days of completing primary series). Dashed line is seropositive threshold (i.e., IgG titer 50.0). Black, unvaccinated; red, mRNA vaccine products; purple, inactivated vaccines; blue, vector‐based vaccines.

The majority of seropositive participants in Phase 2 were vaccinated, with vaccination increasing significantly by age (*p* = 0.001) (Figure S2). Among 364 unvaccinated participants, 283 (77.7%) were seropositive in Phase 2. There were no statistically significant differences in vaccination between men and women (*p* = 0.347). Fully vaccinated participants recorded significantly higher anti‐S titer values than unvaccinated infected participants, regardless of the vaccine product (Figure 2). However, no statistically significant differences were detected between vaccine products that shared the same platform. Based on pairwise comparison of each product, anti‐S IgG titers were highest among participants who had received mRNA vaccines, followed by vector‐based vaccines, and inactivated vaccines (*p* < 0.05).

### 3.2 Longitudinal analysis

Among anti‐N seropositive participants in Phase 1, 82/194 (42.3%) returned at approximately 6 months (April 15–May 25, 2021), and 58/82 (70.7%) anti‐S seropositive participants at the first follow‐up returned at approximately 12 months (November 7, 2021–January 20, 2022) (Figure 2). From baseline, the median elapsed time was 157 days (range 146–194 days) to the first follow‐up and 395 days (range 354–441 days) to the final follow‐up. Loss to follow‐up was higher among younger participants (80.0% in 18–29 years vs. 51.5% in 40–65 years).

**FIGURE 3:**
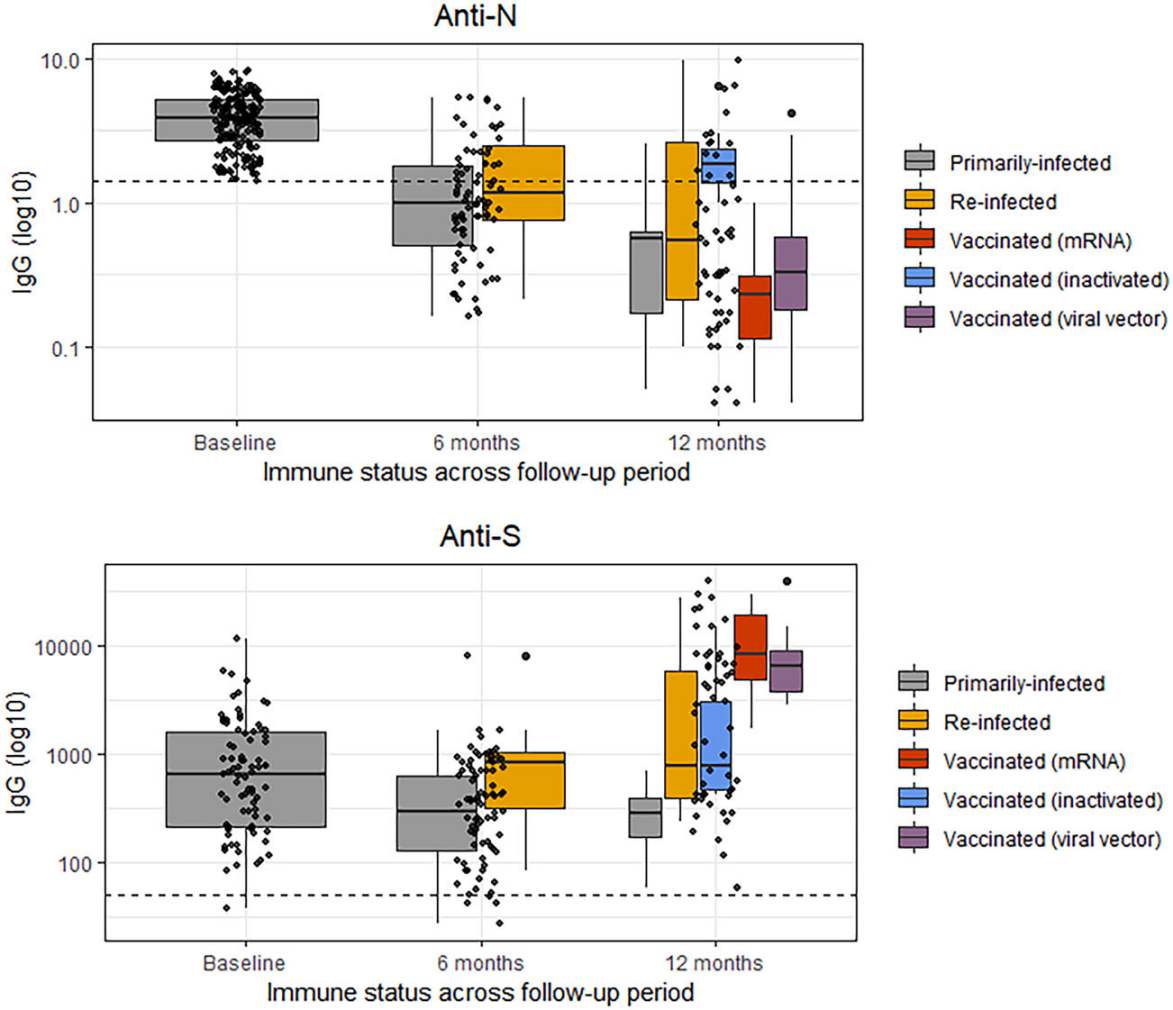
Anti‐N and anti‐S IgG antibody response at baseline, 6 months, and 12 months among primarily infected, re‐infected, and vaccinated blood donors, Sarajevo Canton, BiH, Nov/Dec 2020–Nov 2021/Jan 2022. Primarily infected: unvaccinated participants whose anti‐S and anti‐N IgG titer values decreased over the follow‐up period; re‐infected: unvaccinated participants whose anti‐S and/or anti‐N IgG titer values increased over the follow‐up period; vaccinated: participants who had completed the primary vaccine series. At 6 months, analysis included 64 primarily infected and 16 re‐infected participants; two partially vaccinated participants were excluded. At 12 months, analysis included 11 primarily infected, 12 re‐infected since baseline, and 30 vaccinated participants (11 mRNA [Comirnaty], 8 inactivated [7 Sinopharm, 1 CoronaVac], and 11 viral vector [AstraZeneca]); two partially vaccinated participants and three participants who completed the primary vaccine series >6 months prior were excluded.

No participants had received the COVID‐19 vaccine at baseline. Two participants (2.4%) were partially vaccinated by the first follow‐up, though had not completed the primary vaccine series by the time of serological testing. At the final follow‐up, 30 participants (51.7%) had completed the primary vaccine series within 6 months, excluding 2 (3.4%) who were partially vaccinated and 3 (3.7%) who had completed the primary series more than 6 months prior (Table 3 and Figure 3). Vaccination status was reported verbally.

**TABLE 3:** Characteristics of study population included in longitudinal analysis, Sarajevo Canton, BiH, Nov/Dec 2020–Nov 2021/Jan 2022.

	Baseline (November/December 2020)		6‐month follow‐up (April/May 2021)		12‐month follow‐up (November 2021/January 2022)	
	*N*	%	*N*	%	*N*	%
Overall	194	100.0	82	100.0	58	100.0
Age (years)	Median (range)	35 (18–62)	Median (range)	38 (19–62)	Median (range)	41 (21–62)
18–29	70	36.1	24	29.3	14	24.1
30–39	58	29.9	21	25.6	12	20.7
40–65	66	34.0	37	45.1	32	55.2
Sex						
Male	157	80.9	67	81.7	48	82.8
Female	37	19.1	15	18.3	10	17.2
History of COVID‐19 infection^a^						
Diagnosed	38	19.6	0	0.0	1	1.7
Symptomatic	99	51.0	0	0.0	16	27.6
Hospitalized	2	1.0	0	0.0	0	0.0
Fully vaccinated ≤6 months	0	0.0	0	0.0	30	51.7
Partially vaccinated	–	–	2	2.4	2	3.4
Last dose >6 months prior	–	–	0	0.0	3	5.2
Received booster dose	–	–	0	0.0	0	0.0

^a^ At baseline, participants were asked about infection history within the previous 9 months (since the beginning of the pandemic); at the 6‐month follow‐up, participants were asked about infection history within the previous 6 months (since baseline); and at the 12‐month follow‐up, participants were asked about infection history within the previous 3 months.

At 6 months, 78 (95.1%) participants were seropositive for anti‐S, with median titers decreasing from 665.3 (IQR 216.7–1597.6) to 380.6 (IQR 179.6–801.6); in contrast, 29 (35.4%) participants were seropositive for anti‐N at 6 months, with median titers decreasing from 3.9 (IQR 2.7–5.2) to 1.0 (IQR 0.6–1.8) (Table S1). At 12 months, 58 (100.0%) participants were seropositive for anti‐S, and 14 (24.1%) participants were seropositive for anti‐N, including 6 participants who had received an inactivated vaccine (5 Sinopharm, 1 CoronaVac). No participants reported a history of symptoms or diagnosis at 6 months. At the final follow‐up, 16 participants reported a history of COVID‐19 symptoms in the previous 3 months (median 40 days prior to testing, IQR 29–60), and 1 participant reported diagnosis (80 days prior to testing).

Of 82 participants at the 6‐month follow‐up, 64 (78.0%) were unvaccinated, and anti‐S and anti‐N IgG titer values had not increased since baseline (primarily infected); primarily infected participants declined to 11/58 (19.0%) by the 12‐month follow‐up. Among 11 primarily infected participants by the final follow‐up, all remained seropositive for anti‐S, though anti‐S IgG had declined to 290.8 (IQR 175.7–400.3) from 665.3 (IQR 216.7–1597.6) and anti‐N IgG had declined to 0.6 (IQR 0.2–0.6) from 3.9 (IQR 2.7–5.2) (Figure 3). Vaccinated participants, including those who may have been re‐infected, recorded higher anti‐S IgG titers by the final follow‐up than unvaccinated participants who had likely been re‐infected.

## 4 DISCUSSION

We estimated an adjusted seroprevalence of 17.7% in November and December 2020, which increased significantly to 91.6% among adults aged 18–65 years by November and December 2021, following two additional waves of high SARS‐CoV‐2 transmission and the introduction of COVID‐19 vaccines in Sarajevo Canton, Bosnia and Herzegovina. By the end of 2021, 64.1% of study participants had completed the primary COVID‐19 vaccine series.

Data synthesized from over 800 studies in a recent systematic review and meta‐analysis suggest global seroprevalence of SARS‐CoV‐2 antibodies rose considerably from 5.5% in June 2020 to 45.2% in July 2021, though to just 35.2% among unvaccinated persons.^2^ Results from multivariate modeling suggest higher seroprevalence estimates among low‐ and middle‐ income countries compared to high‐income countries, including in the WHO European Region. Such variation may be due to weaker health system functionality and performance, lower capacity to isolate, and less stringent use of and ability to effectively implement public health and social measures (PHSM).^2^ Authorities in Bosnia and Herzegovina and several other countries in the Western Balkans implemented strict measures early in March 2020. However, measures were met subsequently with a less degree of compliance and new SARS‐CoV‐2 variants emerged, leading to periods of high transmission during 2021 (Figure S1).

The estimated seroprevalence in Phase 1 (17.7%) was significantly higher than reported cumulative incidence based on testing (5.7% by December 31, 2020),^6^ which was not unexpected and has been observed elsewhere. For example, a 2021 systematic review estimated the seroprevalence to be 1.5 to 717 times higher than the cumulative reported case incidence.^11^ Disparities might be explained by testing constraints or the prevalence of asymptomatic, atypical, or pauci‐symptomatic cases.^12^ In Phase 1 of our cross‐sectional analysis, half of seropositive participants reported no history of symptoms associated with COVID‐19, and majority were younger than 40 years. This is consistent with previous studies, which found younger age groups to be more likely to be asymptomatic carriers of SARS‐CoV‐2, less likely to comply with PHSM, and less likely to seek medical attention if clinical symptoms arise.^13^, ^14^ Despite the fact that both phases were conducted following the high SARS‐CoV‐2 transmission wave, participants in Phase 1 reported more COVID‐19 infection history than participants in Phase 2, perhaps as a result of the longer timeframe for reporting history of symptoms.

Vaccination against SARS‐CoV‐2 significantly contributed to high seroprevalence during Phase 2 of our study. Global studies have demonstrated the critical role of vaccination in achieving population immunity to SARS‐CoV‐2, primarily to protect against severe disease.^12^, ^15^ A study conducted in the United States among blood donors estimated that seroprevalence increased from 3.5% to 11.5% between July and December 2020 and then to 83.3% by May 2021 as a result of infection‐ and vaccine‐induced antibody response, with the majority of the seropositivity attributable to vaccination.^16^ Similarly, a study in Geneva, Switzerland, conducted in July 2021, found a population seroprevalence of 66.1%, where 36.2% had developed antibodies due to vaccination.^17^

We found that fully vaccinated participants (including those who might have previously been infected) recorded significantly higher anti‐S titer values than unvaccinated, infected participants, regardless of the vaccine product, though titers varied across vaccine platforms. We also observed a higher antibody response among vaccinated persons who had a prior infection. A systematic review and meta‐analysis based on three vaccine efficacy trials and four observational studies from the United States, Israel, and the United Kingdom found no significant difference in the overall level of protection following infection versus vaccination; included studies spanned the period prior to and following the emergence of the Delta variant.^18^ In contrast, observational studies found a history of prior infection provided greater protection against subsequent infection compared to vaccination alone; however, the overall risk of re‐infection was lowest among those who were vaccinated following infection (hybrid immunity) during periods of Delta predominance.^19^, ^20^

Anti‐S IgG titers among seropositive participants in our study remained stable over 12 months. This finding is consistent with previous research among confirmed COVID‐19 cases from 6 to 12 months following infection, though antibody response following infection was highly heterogeneous between individuals.^21^, ^22^, ^23^, ^24^, ^25^, ^26^, ^27^ Previous studies also found anti‐N antibody response wanes earlier, becoming undetectable in most cases by 5 to 7 months.^28^, ^29^ Although the duration of immunity may depend on the type of vaccine, anti‐S antibodies remain detectable at least 6 to 8 months following vaccination.^30^ However, greater waning in vaccine effectiveness was observed in persons 65 years of age or older.^31^

A limitation of our study is that our sample consisted of blood donors, a generally healthy population that may not be representative of the general population. Most study participants were aged younger than 40 years (64.7%) and male (70.0%), resulting in differences in the age‐ and sex‐ distribution between the study population and Sarajevo Canton. Additionally, there was a higher rate of non‐responses in Phase 2 of our study. We relied on self‐reported history of SARS‐CoV‐2 infection and for some participants on vaccination status, which may not be accurate. Individuals who experienced COVID‐19 symptoms within 14 days were excluded, which may have underestimated the prevalence; however, this is a requirement for donating blood in FBiH. The study population is restricted to residents of Sarajevo Canton, and results may not be generalizable to other geographic areas or Bosnia and Herzegovina as a whole. In addition, we may have underestimated seroprevalence due to waning antibodies, particularly in Phase 1, during which we relied on anti‐N titers to determine seropositivity due to lack of capacity to test for anti‐S antibody response at that time. Anti‐N titers are known to decline earlier compared to anti‐S titers.^32^ Few participants in our study reported severe disease, with hospitalization reported by just three participants in Phase 1 and one participant in Phase 2, so it was not possible to assess an association with antibody response. Finally, the manufacturer's cut‐off threshold of the employed test may lead to the underestimating of seroprevalence, as suggested in certain peer‐reviewed papers.^33^, ^34^

This study has many strengths. Our study addresses an important gap in COVID‐19 surveillance in Bosnia and Herzegovina and contributes evidence from the Western Balkans, a region that has reported among the highest mortality associated with COVID‐19.^35^ We used validated tests and adjusted for the age and sex distribution in the population of Sarajevo Canton. We also estimated durability of two types of antibodies (anti‐N and anti‐S), which are known to have different periods of detectability.^36^, ^37^, ^38^, ^39^ We also monitored the evolution of seroprevalence to SARS‐CoV‐2 following its introduction to a well‐defined geographic area and present vaccination data across three COVID‐19 vaccine platforms. Finally, use of the standardized WHO UNITY protocol allows for comparison and synthesis of our study results with other studies globally.

This study was conducted prior to the widespread circulation of the SARS‐CoV‐2 variant of concern, Omicron (Figure S1), in Sarajevo Canton and within 9 months of the introduction of COVID‐19 vaccines. Evidence of waning protection from previous infection and vaccination highlights the importance of booster doses of vaccines against COVID‐19.^31^, ^40^, ^41^, ^42^, ^43^

## 5 CONCLUSION

We found the number of reported COVID‐19 cases based on testing significantly underestimated the number of individuals who were infected with SARS‐CoV‐2 in Sarajevo Canton. Over the 2‐year pandemic period, the majority of the population of Sarajevo Canton developed infection‐ or vaccine‐induced antibodies against SARS‐CoV‐2, even prior to the surge in infections caused by the Omicron variant, which may suggest some level of population protection against severe disease during future surges. Further efforts should continue to broaden vaccination coverage, especially in vulnerable populations, and implement strategic use of booster doses.

## AUTHOR CONTRIBUTIONS

*Conceptualization*: Sanjin Musa, Isabel Bergeri and Richard Pebody. *Data curation*: Sanjin Musa, Elma Catovic Baralija, and Veronica Ivey Sawin. *Formal analysis*: Veronica Ivey Sawin. *Funding acquisition*: Mirza Palo, Sanjin Musa, Isabel Bergeri, and Richard Pebody. *Investigation*: Sanjin Musa, Elma Catovic Baralija, Gorana Ahmetovic‐Karic, Alma Ljuca, Sanela Dostovic‐Halilovic, and Rozalija Nedic. *Methodology*: Sanjin Musa, Veronica Ivey Sawin, Aisling Vaughan, Isabel Bergeri and Anthony Nardone. *Project administration*: Sanjin Musa, Elma Catovic Baralija, Aisling Vaughan, Mirza Palo, and Sinisa Skocibusic. *Resources*: Sanjin Musa, Elma Catovic Baralija, Aisling Vaughan, Mirza Palo, Richard Pebody, and Isabel Bergeri. *Software*: Veronica Ivey Sawin. *Supervision*: Sanjin Musa, Elma Catovic Baralija, Sinisa Skocibusic, Seila Cilovic Lagarija, Aisling Vaughan, and Richard Pebody. *Validation*: Veronica Ivey Sawin. *Visualization*: Sanjin Musa, Veronica Ivey Sawin, and Anthony Nardone. *Writing—original draft preparation*: Sanjin Musa, Veronica Ivey Sawin, Aisling Vaughan, and Anthony Nardone. *Writing—review and editing*: Sanjin Musa, Elma Catovic Baralija, Veronica Ivey Sawin, Anthony Nardone, Mirza Palo, Sinisa Skocibusic, Mia Blazevic, Seila Cilovic Lagarija, Gorana Ahmetovic‐Karic, Alma Ljuca, Sanela Dostovic‐Halilovic, Rozalija Nedic, Lorenzo Subissi, Rawi Ibrahim, Golubinka Boshevska, Isabel Bergeri, Richard Pebody, and Aisling Vaughan.

## CONFLICT OF INTEREST STATEMENT

VIS has served as a consultant for WHO Country Office in Bosnia and Herzegovina. Co‐authors report no conflicts of interest.

### PEER REVIEW

The peer review history for this article is available at https://www.webofscience.com/api/gateway/wos/peer-review/10.1111/irv.13182.

## ETHICS STATEMENT

The protocols for each phase of this study were reviewed and approved by the Ethics Review Committee at the Institute for Public Health of the Federation of Bosnia and Herzegovina. The WHO Ethics Review Committee reviewed and approved the protocol for Phase 2 of the study. The protocol is registered with clinicaltrials.gov (NCT05124535) and study results are reported according to the STROBE statement. Participation in the study was voluntary and all participants provided informed consent.

## Supporting information


**Figure S1.** Confirmed COVID‐19 cases in Sarajevo Canton and study periods. A timeline of study recruitment and follow‐up periods is shown alongside official confirmed cases from the beginning of March 2020 to the end of January 2022. Participants were recruited for Phase 1 between November 7–December 2, 2020, and for Phase 2 between November 7–December 31, 2021 (blue shade). In the longitudinal analysis, seropositive participants were reassessed at 6 months April 15–May 25, 2021, and 12 months November 7, 2021–January 20, 2022 (gray shade). COVID‐19 vaccination began in Sarajevo Canton in March 2021.
**Figure S2**. Crude seroprevalence across types of immunity by vaccination status and age groups, Sarajevo Canton, BiH, Nov/Dec 2021.
**Table S1**. Immune durability of anti‐N and anti‐S IgG titers at 6‐ and 12‐months.

## Data Availability

De‐identified data and R script will be made available upon request.

## References

[1] World Health Organization. Statement on the second meeting of the International Health Regulations . (2005) Emergency Committee regarding the outbreak of novel coronavirus (2019‐nCoV). https://www.who.int/news/item/30‐01‐2020‐statement‐on‐the‐second‐meeting‐of‐the‐international‐health‐regulations‐(2005)‐emergency‐committee‐regarding‐the‐outbreak‐of‐novel‐coronavirus‐(2019‐ncov)

[2] Bergeri I , Whelan M , Ware H , et al. Global epidemiology of SARS‐CoV‐2 infection: a systematic review and meta‐analysis of standardized population‐based seroprevalence studies, Jan 2020‐Dec 2021. Prescriber. doi:10.1101/2021.12.14.21267791

[3] Institute for Statistics of the Federation of Bosnia and Herzegovina . Statistical Yearbook of the Federation of Bosnia and Herzegovina 2021. http://fzs.ba/index.php/publikacije/statisticki-godisnjaciljetopisi/

[4] World Health Organization Regional Office for Europe . COVID‐19 situation in the WHO European region. 2021. https://who.maps.arcgis.com/apps/dashboards/ead3c6475654481ca51c248d52ab9c61

[5] Sarajevo Canton Government . Vaccination against COVID‐19 in Sarajevo Canton. 2021. https://vlada.ks.gov.ba/aktuelnosti/novosti-vezane-za-koronu-virus/u-kantonu-sarajevo-protiv-koronavirusa-vakcinisano-3

[6] Institute for Public Health of the Federation of Bosnia and Herzegovina . Overview of the epidemiological situation of COVID‐19. 2022. https://www.zzjzfbih.ba/pregled-epidemioloske-situacije-covid-19/

[7] Murhekar MV , Clapham H . COVID‐19 serosurveys for public health decision making. Lancet Glob Health. 2021;9(5):e559‐e560. doi:10.1016/S2214-109X(21)00057-7 33705691 PMC8049585

[8] Wu SL , Mertens AN , Crider YS , et al. Substantial underestimation of SARS‐CoV‐2 infection in the United States. Nat Commun. 2020;11(1):4507. doi:10.1038/s41467-020-18272-4 32908126 PMC7481226

[9] Pollán M , Pérez‐Gómez B , Pastor‐Barriuso R , et al. Prevalence of SARS‐CoV‐2 in Spain (ENE‐COVID): a nationwide, population‐based seroepidemiological study. Lancet. 2020;396(10250):535‐544. doi:10.1016/S0140-6736(20)31483-5 32645347 PMC7336131

[10] World Health Organization . Population‐based age‐stratified seroepidemiological investigation protocol for coronavirus 2019 (COVID‐19) infection. 2020. https://www.who.int/publications-detail-redirect/WHO-2019-nCoV-Seroepidemiology-2020.2

[11] Byambasuren O , Dobler CC , Bell K , et al. Comparison of seroprevalence of SARS‐CoV‐2 infections with cumulative and imputed COVID‐19 cases: systematic review. PLoS ONE. 2021;16(4):e0248946. doi:10.1371/journal.pone.0248946 33798211 PMC8018669

[12] Byambasuren O , Cardona M , Bell K , Clark J , McLaws ML , Glasziou P . Estimating the extent of asymptomatic COVID‐19 and its potential for community transmission: systematic review and meta‐analysis. Off J Assoc Med Microbiol Infect Dis Can. 2020;5(4):223‐234. doi:10.3138/jammi-2020-0030 PMC9602871 36340059

[13] Boehmer TK . Changing age distribution of the COVID‐19 pandemic—United States, May–August 2020. MMWR Morb Mortal Wkly Rep. 2020;69(39). doi:10.15585/mmwr.mm6939e1 PMC7537561 33001872

[14] Hutchins HJ . COVID‐19 mitigation behaviors by age group—United States, April–June 2020. MMWR Morb Mortal Wkly Rep. 2020;69(43). doi:10.15585/mmwr.mm6943e4 PMC7641002 33119562

[15] Brown CM , Vostok J , Johnson H , et al. Outbreak of SARS‐CoV‐2 infections, including COVID‐19 vaccine breakthrough infections, associated with large public gatherings ‐ Barnstable County, Massachusetts, July 2021. MMWR Morb Mortal Wkly Rep. 2021;70(31):1059‐1062. doi:10.15585/mmwr.mm7031e2 34351882 PMC8367314

[16] Jones JM , Stone M , Sulaeman H , et al. Estimated US infection‐ and vaccine‐induced SARS‐CoV‐2 seroprevalence based on blood donations, July 2020‐May 2021. Jama. 2021;326(14):1400‐1409. doi:10.1001/jama.2021.15161 34473201 PMC8414359

[17] Stringhini S , Zaballa ME , Pullen N , et al. Seroprevalence of anti‐SARS‐CoV‐2 antibodies 6 months into the vaccination campaign in Geneva, Switzerland, 1 June to 7 July 2021. Euro Surveill Bull Eur Sur Mal Transm Eur Commun Dis Bull. 2021;26(43). doi:10.2807/1560-7917.ES.2021.26.43.2100830 PMC8555371 34713799

[18] Shenai MB , Rahme R , Noorchashm H . Equivalency of protection from natural immunity in COVID‐19 recovered versus fully vaccinated persons: a systematic review and pooled analysis. Cureus. 2021;13(10):e19102. doi:10.7759/cureus.19102 34868754 PMC8627252

[19] Gazit S , Shlezinger R , Perez G , et al. Comparing SARS‐CoV‐2 natural immunity to vaccine‐induced immunity: reinfections versus breakthrough infections. Published online August 25, 2021:2021.08.24.21262415. doi:10.1101/2021.08.24.21262415

[20] Murugesan M , Mathews P , Paul H , Karthik R , Mammen JJ , Rupali P . Protective effect conferred by prior infection and vaccination on COVID‐19 in a healthcare worker cohort in South India. PLoS ONE. 2021;17(5):e0268797. doi:10.2139/ssrn.3914633 PMC9122209 35594270

[21] Masiá M , Fernández‐González M , Telenti G , et al. Durable antibody response one year after hospitalization for COVID‐19: a longitudinal cohort study. J Autoimmun. 2021;123:102703. doi:10.1016/j.jaut.2021.102703 34303083 PMC8289631

[22] Dan JM , Mateus J , Kato Y , et al. Immunological memory to SARS‐CoV‐2 assessed for up to 8 months after infection. Science. 2021;371(6529):eabf4063. doi:10.1126/science.abf4063 33408181 PMC7919858

[23] Li C , Yu D , Wu X , et al. Twelve‐month specific IgG response to SARS‐CoV‐2 receptor‐binding domain among COVID‐19 convalescent plasma donors in Wuhan. Nat Commun. 2021;12(1):4144. doi:10.1038/s41467-021-24230-5 34230476 PMC8260809

[24] Chia WN , Zhu F , Ong SWX , et al. Dynamics of SARS‐CoV‐2 neutralising antibody responses and duration of immunity: a longitudinal study. Lancet Microbe. 2021;2(6):e240‐e249. doi:10.1016/S2666-5247(21)00025-2 33778792 PMC7987301

[25] Anand SP , Prévost J , Nayrac M , et al. Longitudinal analysis of humoral immunity against SARS‐CoV‐2 Spike in convalescent individuals up to 8 months post‐symptom onset. Cell Rep Med. 2021;2(6):100290. doi:10.1016/j.xcrm.2021.100290 33969322 PMC8097665

[26] He Z , Ren L , Yang J , et al. Seroprevalence and humoral immune durability of anti‐SARS‐CoV‐2 antibodies in Wuhan, China: a longitudinal, population‐level, cross‐sectional study. The Lancet. 2021;397(10279):1075‐1084. doi:10.1016/S0140-6736(21)00238-5 PMC7972311 33743869

[27] Alfego D , Sullivan A , Poirier B , et al. A population‐based analysis of the longevity of SARS‐CoV‐2 antibody seropositivity in the United States. eClinicalMedicine. 2021;36:36, 100902. doi:10.1016/j.eclinm.2021.100902 PMC8143650 34056568

[28] Ripperger TJ , Uhrlaub JL , Watanabe M , et al. Orthogonal SARS‐CoV‐2 serological assays enable surveillance of low‐prevalence communities and reveal durable humoral immunity. Immunity. 2020;53(5):925‐933.e4. doi:10.1016/j.immuni.2020.10.004 33129373 PMC7554472

[29] Wheatley AK , Juno JA , Wang JJ , et al. Evolution of immune responses to SARS‐CoV‐2 in mild‐moderate COVID‐19. Nat Commun. 2021;12(1):1162. doi:10.1038/s41467-021-21444-5 33608522 PMC7896046

[30] Campillo‐Luna J , Wisnewski AV , Redlich CA . Human IgG and IgA responses to COVID‐19 mRNA vaccines. Published online March 26, 2021:2021.03.23.21254060. doi:10.1101/2021.03.23.21254060 PMC8208542 34133415

[31] Andrews N , Stowe J , Kirsebom F , et al. COVID‐19 vaccine effectiveness against the Omicron (B.1.1.529) variant. N Engl J Med. 2022;386(16):1532‐1546. doi:10.1056/NEJMoa2119451 35249272 PMC8908811

[32] Fenwick C , Croxatto A , Coste AT , et al. Changes in SARS‐CoV‐2 antibody responses impact the estimates of infections in population‐based seroprevalence studies. 2020. 10.1101/2020.07.14.20153536 PMC7925109 33144321

[33] Mohanraj D , Bicknell K , Bhole M , Webber C , Taylor L , Whitelegg A . Antibody responses to SARS‐CoV‐2 infection‐comparative determination of seroprevalence in two high‐throughput assays versus a sensitive spike protein ELISA. Vaccines (Basel). 2021;9(11):1310. doi:10.3390/vaccines9111310 34835241 PMC8624239

[34] Barchuk A , Shirokov D , Sergeeva M , et al. Evaluation of the performance of SARS‐CoV‐2 antibody assays for a longitudinal population‐based study of COVID‐19 spread in St. Petersburg, Russia. J Med Virol. 2021;93(10):5846‐5852. doi:10.1002/jmv.27126 34081328 PMC8242745

[35] Johns Hopkins University . Mortality Analyses. Johns Hopkins Coronavirus Resource Center; 2022. https://coronavirus.jhu.edu/data/mortality

[36] Isho B , Abe KT , Zuo M , et al. Persistence of serum and saliva antibody responses to SARS‐CoV‐2 spike antigens in COVID‐19 patients. Sci Immunol. 2020;5(52):eabe5511. doi:10.1126/sciimmunol.abe5511 33033173 PMC8050884

[37] Bolotin S , Tran V , Osman S , et al. SARS‐CoV‐2 seroprevalence survey estimates are affected by anti‐nucleocapsid antibody decline. J Infect Dis. 2021;223(8):1334‐1338. doi:10.1093/infdis/jiaa796 33400794 PMC7928877

[38] Deeks JJ , Dinnes J , Takwoingi Y , et al. Antibody tests for identification of current and past infection with SARS‐CoV‐2. Cochrane Database Syst Rev. 2020;(6):CD013652. doi:10.1002/14651858.CD013652 32584464 PMC7387103

[39] Bastos ML , Tavaziva G , Abidi SK , et al. Diagnostic accuracy of serological tests for covid‐19: systematic review and meta‐analysis. BMJ. 2020;370:m2516. doi:10.1136/bmj.m2516 32611558 PMC7327913

[40] Hall V , Foulkes S , Insalata F , et al. Protection against SARS‐CoV‐2 after Covid‐19 vaccination and previous infection. N Engl J Med. 2022;386(13):1207‐1220. doi:10.1056/NEJMoa2118691 35172051 PMC8908850

[41] Goldberg Y , Mandel M , Bar‐On YM , et al. Protection and waning of natural and hybrid immunity to SARS‐CoV‐2. N Engl J Med. 2022;386(23):2201‐2212. doi:10.1056/NEJMoa2118946 35613036 PMC9165562

[42] Menni C , May A , Polidori L , et al. COVID‐19 vaccine waning and effectiveness and side‐effects of boosters: a prospective community study from the ZOE COVID study. Lancet Infect Dis. 2022;22(7):1002‐1010. doi:10.1016/S1473-3099(22)00146-3 35405090 PMC8993156

[43] Altarawneh HN , Chemaitelly H , Ayoub HH , et al. Effects of previous infection and vaccination on symptomatic omicron infections. N Engl J Med. 2022;387(1):21‐34. doi:10.1056/NEJMoa2203965 35704396 PMC9258753

