# Supplementary material for: Evolution of seroprevalence to SARS‐CoV‐2 in blood donors in Sarajevo Canton, Federation of Bosnia and Herzegovina: Cross‐sectional and longitudinal studies
Source: Influenza Other Respir Viruses. 2023 Aug 22;17(8):e13182. doi: 10.1111/irv.13182 (PMC10444603; doi:10.1111/irv.13182)
Supplement: Supplementary file 1 — Figure S1. Confirmed COVID‐19 cases in Sarajevo Canton and study periods. A timeline of study recruitment and follow‐up periods is shown alongside official confirmed cases from the beginning of March 2020 to the end of January 2022. Participants were recruited for Phase 1 between November 7–December 2, 2020, and for Phase 2 between November 7–December 31, 2021 (blue shade). In the longitudinal analysis, seropositive participants were reassessed at 6 months April 15–May 25, 2021, and 12 months November 7, 2021–January 20, 2022 (gray shade). COVID‐19 vaccination began in Sarajevo Canton in March 2021. Figure S2. Crude seroprevalence across types of immunity by vaccination status and age groups, Sarajevo Canton, BiH, Nov/Dec 2021. Table S1. Immune durability of anti‐N and anti‐S IgG titers at 6‐ and 12‐months. [file IRV-17-e13182-s001.docx]

## SUPPORTING INFORMATION


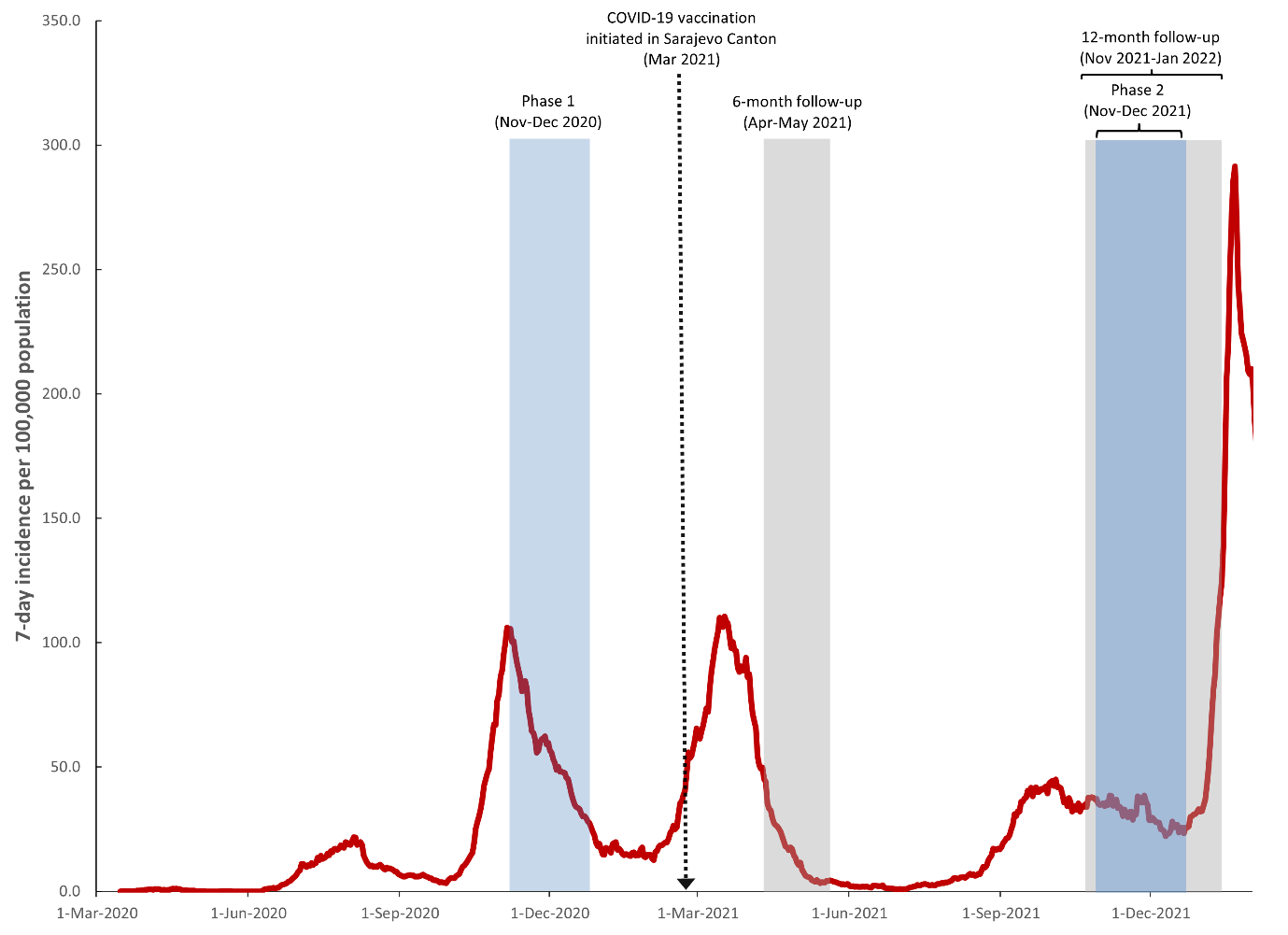


**Figure S1. Confirmed COVID-19 cases in Sarajevo Canton and study periods.** A timeline of study recruitment and follow-up periods is shown alongside official confirmed cases from the beginning of March 2020 to the end of January 2022. Participants were recruited for Phase 1 between November 7-December 2, 2020, and for Phase 2 between November 7-December 31, 2021 (blue shade). In the longitudinal analysis, seropositive participants were reassessed at 6 months April 15-May 25, 2021, and 12 months November 7, 2021-January 20, 2022 (grey shade). COVID-19 vaccination began in Sarajevo Canton in March 2021.


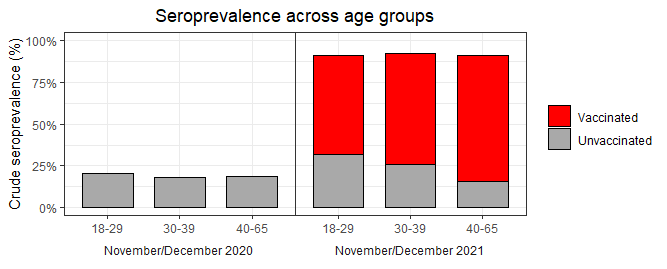


**Figure S2. Crude seroprevalence across types of immunity by vaccination status and age groups, Sarajevo Canton, BiH, Nov/Dec 2021.**

**Table S1. Immune durability of anti-N and anti-S IgG titers at 6- and 12-months**

|  | **Baseline**  **(November/December 2020)** | | **6-month follow-up**  **(April/May 2021)** | | **12-month follow-up**  **(November 2021/January 2022)** | | |  |
| --- | --- | --- | --- | --- | --- | --- | --- | --- |
|  | **N (%)** | **Median IgG (IQR)** | **N (%)** | **Median IgG (IQR)** | **N (%)** | **Median IgG (IQR)** |  |  |
| **Overall** | 1015 (100.0) | -- | 82 (100.0) | -- | 58 (100.0) | -- |  |  |
| Primarily- infected | 194 (100.0 | -- | 64 (78.0) | -- | 11 (18.9) | -- |  |  |
| Re-infected | -- | -- | 16 (19.5) | -- | 12 (20.7) | -- |  |  |
| Vaccinated | 0 (0.0) | -- | 0 (0.0) | -- | 30 (55.2) | -- |  |  |
| mRNA | -- | -- | -- | -- | 11 (19.0) | -- |  |  |
| Inactivated | -- | -- | -- | -- | 8 (13.8) | -- |  |  |
| Viral vector | -- | -- | -- | -- | 11 (19.0) | -- |  |  |
| **Anti-N IgG+** | 194 (19.1) | 3.9 (2.4) | 29 (35.4) | 1.0 (1.2) | 14 (24.1) | 0.3 (1.1) |  |  |
| Primarily- infected | 194 (100.0) | 3.9 (2.4) | 21 (32.8) | 1.0 (1.3) | 2 (18.2) | 0.6 (0.5) |  |  |
| Re-infected | -- | -- | 8 (0.5) | 1.2 (1.8) | 1 (8.3) | 0.6 (2.4) |  |  |
| Vaccinated | -- | -- | -- | -- | 8 (26.7) | 0.4 (1.2) |  |  |
| mRNA | -- | -- | -- | -- | 0 (0.0) | 0.2 (0.2) |  |  |
| Inactivated | -- | -- | -- | -- | 6 (75.0) | 1.9 (1.0) |  |  |
| Viral vector | -- | -- | -- | -- | 2 (18.2) | 0.3 (0.4) |  |  |
| **Anti-S IgG+** | 81 (98.8)* | 665.3 (1380.9) | 78 (95.1) | 373.9 (636.3) | 58 (100.0) | 2936.0 (6852.3) |  |  |
| Primarily- infected | 81 (98.8)* | 665.3 (1380.9) | 60 (93.8) | 292.1 (511.3) | 11 (100.0) | 290.8 (224.6) |  |  |
| Re-infected | -- | -- | 16 (100.0) | 852.5 (725.2) | 12 (100.0) | 807.1 (5512.7) |  |  |
| Vaccinated | -- | -- | -- | -- | 30 (100.0) | 5900.0 (6499.1) |  |  |
| mRNA | -- | -- | -- | -- | 11 (100.0) | 8565.1 (14479.2) |  |  |
| Inactivated | -- | -- | -- | -- | 8 (100.0) | 856.4 (2885.6) |  |  |
| Viral vector | -- | -- | -- | -- | 11 (100.0) | 6616.7 (5107.7) |  |  |
| *Anti-S IgG was retroactively assessed using stored aliquots only for those included in the 6-month follow-up (n=82). Anti-N IgG+ if ≥1.4; anti-S IgG+ if ≥ 50.0. mRNA vaccines (11 Comirnaty); inactivated (7 Sinopharm, 1 CoronaVac); and viral vector (11 AstraZeneca). | | | | | | | | |
